# Response of the primary tumor in symptomatic and asymptomatic stage IV colorectal cancer to combined interventional endoscopy and palliative chemotherapy

**DOI:** 10.1186/1471-2407-9-218

**Published:** 2009-07-01

**Authors:** Silke Cameron, Diana Hünerbein, Tümen Mansuroglu, Thomas Armbrust, Jens-Gerd Scharf, Harald Schwörer, László Füzesi, Giuliano Ramadori

**Affiliations:** 1 Department of Gastroenterology and Endocrinology, University Clinic of the Georg August University, Göttingen, Germany; 2 Department of Gastroenteropathology, University Clinic of the Georg August University, Göttingen, Germany

## Abstract

**Background:**

The treatment of the primary tumor in advanced metastatic colorectal cancer (CRC) is still a matter of discussion. Little attention has thus far been paid to the endoscopically observable changes of the primary in non-curatively resectable stage IV disease.

**Methods:**

20 patients [14 men, 6 women, median age 67 (39–82) years] were observed after initial diagnosis of non-curatively resectable metastasized symptomatic (83%) or asymptomatic (17%) CRC, from June 2002 to April 2009. If necessary, endoscopic tumor debulking was performed. 5-FU based chemotherapy was given immediately thereafter. In 10 patients, chemotherapy was combined with antibody therapy.

**Results:**

Response of the primary was observed in all patients. Local symptoms were treated endoscopically whenever necessary (obstruction or bleeding), and further improved after chemotherapy was started: Four patients showed initial complete endoscopic disappearance of the primary. In an additional 6 patients, only adenomatous tissue was histologically detected. In both these groups, two patients revealed local tumor relapse after interruption of therapy. Local tumor regression or stable disease was achieved in the remaining 10 patients. 15 patients died during the observation time. In 13 cases, death was related to metastatic disease progression. The mean overall survival time was 19.6 (3–71) months. No complications due to the primary were observed.

**Conclusion:**

This study shows that modern anti-cancer drugs combined with endoscopic therapy are an effective and safe treatment of the symptomatic primary and ameliorate local complaints without the need for surgical intervention in advanced UICC stage IV CRC.

## Background

Metastasizing colorectal cancer (CRC) is one of the leading causes of tumor-related death [1]. At the time of diagnosis, about 20% of the tumors have synchronous non-resectable liver metastases [2,3]. In these patients, the aim of treatment is to prolong survival and to maintain an acceptable quality of life. Modern chemotherapy may serve this purpose [2,4]. Depending on the regimen used, response rates vary between 20–70% [5,6]. Most phase III trials using combination therapy have reported a response rate on the order of 40% [7]. In 20–30%, stability of the metastases can be achieved [8,9]. Mean survival time in patients with advanced stage CRC varies between 12–21 months after diagnosis [10].

With the development of modern chemotherapy and endoscopic techniques, new therapeutical options for the treatment of advanced metastasized CRC have emerged. The 5-FU-leucovorin-based regimen combined with oxaliplatin or irinotecan was approved for the first-line treatment of advanced CRC and has become the treatment standard [11]. Combination with antibody therapy, such as the anti-VEGF-antibody bevacizumab [8] or the anti-EGFR-antibody cetuximab have become further options [12].

However, surgical treatment of the primary tumor in non-curatively resectable stage IV disease is still a matter of discussion [13-15]. In the past, investigators have recommended elective resection of the primary tumor to prevent the need for urgent surgical procedures arising from local complications [16]. More recently, some authors have suggested elective resection of asymptomatic colorectal cancers at least in a subset of patients with less advanced stage IV disease [17,18]. Others have suggested deferring the resection of minimally symptomatic CRC, as in most of these patients death is related to systemic disease progression rather than complications related to the primary lesion [2,19]. In the setting of incurable stage IV CRC, in which colonic resection has further been associated with a high mortality rate [19,20] no survival advantage is gained by resection of an asymptomatic primary lesion [21].

As metastatic disease progression determines the outcome of these patients, efforts have been concentrated on the surveillance of tumor-related metastasis, typically measured by imaging techniques at scheduled intervals [22-24]. In case of non-resected asymptomatic CRC, surveillance does generally not include colonoscopies at tight intervals. Local inspection of the primary may be repeated to select patients for operation from a primarily non-operative setting.

Encouraging preliminary studies [14,25,26] prompted us to observe the local tumor behaviour in patients with non-curatively resectable stage IV CRC during chemotherapy treatment. We are aware that modern treatment of these patients is based on individual treatment concepts which need to be re-evaluated during the course of therapy. Sometimes, resection of metastasis is possible so that palliative chemotherapy becomes a later therapy option. We here report on the local tumor behaviour, the time-to-progression of the primary and the overall survival of 20 patients treated with modern pharmacotherapy with or without endoscopic tumor debulking.

### Patients

For the current study, all the 23 patients with unoperated primary tumors and unresectable metastatic UICC stage IV CRC seen in the Department of Gastroenterology, University Clinic Goettingen, between June 2002 and June 2008 were considered for inclusion. Patients had to be able and willing to receive chemotherapy with – if necessary – local tumor debulking. Patients with significant cardiovascular disease or inadequate hematologic parameters were excluded. Of the 23 patients, the following three patients were not included in this study: One 83 year old female patient declined chemotherapeutic treatment, an 80 year old female patient had initially been operated and now presented with local recurrence, and one 62 year old male patient died of an infection after replacement of the tricuspid valve shortly after the cancer was diagnosed. The remaining 20 patients form our study collective and were observed until April 2009. Table 1 summarises the patients' characteristics.

**Table 1:** Tumor characteristics at baseline in patients with advanced stage IV CRC.

	No. of patients n = 20	%
**Age (Years)**	67 (39–82)	
Median (range)		

**Sex**		
Male	14	70%
Female	6	30%

**Tumor origin**		
Colon	14	70%
Rectum	6	30%

**Performance status (ECOG)**		
0	1	5%
1	12	60%
2	5	25%
3	2	10%
4	0	0%

**Sites of synchrone metastases**		
Liver	17	85%
Lung	11	55%
Lymph nodes	5	25%
Peritoneal	2	10%
Bone	1	5%

**Number of metastatic sites**		
1	8	40%
2	6	30%
≥3	6	30%

Data collection and publication of the data was approved by the local ethics committee. All patients were chemotherapy naïve at the beginning of this study and above 18 years of age. They had adequate hematologic parameters (absolute neutrophil count ≥ 1–5 × 10^3^/μl and platelets ≥ 100 × 10^3^/μl), creatinine and total bilirubin < 2.25 times the upper limit of normal and absence of active infection.

## Methods

Tumor staging was undertaken before therapy and every three to four months after the beginning of treatment. After the initial staging and a possible initial tumor debulking, the patients underwent chemotherapy treatment.

Endoscopic tumor debulking of the primary tumor was performed if the tumor mass resulted in a luminal obstruction of about 80% or above as detected endoscopically during staging, or if bleeding or obstruction symptoms were reported. The endoscopic treatment was done either by using a standard polypectomy snare technique alone or in combination with argon plasma coagulation. If the metastasis was considered operable during ongoing treatment, operation of both the metastasis and of the primary was offered.

Staging included the measurement of the tumor marker CEA (carcino embryonic antigen) and the imaging of the metastasis (CT scan or ultrasound) and, if considered necessary, the inspection of the local tumor by sigmoidoscopy or colonoscopy. Endoscopy was initially performed every three to four months in non-symptomatic patients. In symptomatic patients, endoscopic tumor debulking was performed at, on average, two sessions (range 1–5) in the first two months.

Since endoscopic examinations can be associated with complications, we comment that there is a question as to how often they should be performed. For the patients in this study, the high degree of luminal obstruction initially suggested regular examination and where appropriate, debulking.

*Complete response of the primary *was defined as macroscopically intact mucosa and histologically confirmed absence of malignant cells in the biopsy.

*Partial response of the primary *was defined as macroscopic tumor regression with histologically confirmed tumor cells.

Endoscopic regression of the primary has not previously been defined in the literature. The inspection of the gut lumen was done by experienced endoscopists (TA, JGS, HS, GR), and included the description of bowel motility and the possibility of passage of the tumor with a colonoscope. Depending on the macroscopic appearance, the decision had to be taken whether endoscopic intervention was needed. Complete luminal occlusion – no passage possible with the thinnest endoscope (4 mm) – was rated 100%. If the tumor could just be passed with the colonoscope (14 mm), luminal occlusion was considered to be 85%. We are of course aware this is a rather subjective method.

### Statistical considerations

The primary objective of this study was to evaluate the response of the primary tumor to up-to-date anti-cancer therapy. Time-to-progression of the primary and overall survival were estimated by the Kaplan-Meier method. Data are expressed as mean, median (range) and per cent. Calculations were performed with SPSS™ (version 13) and SigmaPlot™ (version 10).

## Results

### Patients

Twenty patients [14 men, 6 women, median age 67 (39–82) years] with non-resectable UICC stage IV CRC (14 colon, 6 rectum) at diagnosis were included from June 2002 to June 2008 and documented until April 2009. The obtained data were retrospectively analysed. Eastern Cooperative Oncology Group Performance Status (ECOG) before initiation of the therapy was 0 or 1 in one and twelve patients, respectively (65%) and ≥ 2 in seven patients (35%). The majority of the patients (12/20) had more than one site of synchronous metastases. Liver metastases were predominant and present in 17 patients (85%). Other sites of metastasis included, in decreasing frequency of occurrence, lung, lymph nodes and peritoneum as well as bone (Table 1).

### Treatment Administration

In 17 of the 20 patients (85%), endoscopic tumor debulking was performed prior to and, if necessary, in parallel with chemotherapy. In 3 patients, stool passage was re-established [27]. In another 4 patients, repeated bleeding was stopped.

The patients were then subjected to a modern chemotherapy regimen either starting with FOLFOX (folinic acid, 5-fluorouracil (FU), oxaliplatin) in 15 patients or with FOLFIRI (folinic acid, 5-FU, irinotecan) in three patients. One patient started with Xeliri and another patient (82 years) with a bad performance status (ECOG 3) initially received the Ardalan protocol (folinic acid, 5-FU). In 50% of the 20 cases, chemotherapy was combined with antibody therapy. Nine of these patients received bevacizumab (Avastin), one patient received cetuximab (Erbitux).

In 13 patients, chemotherapy was started within the first week after tumor diagnosis. In the remaining patients (7/20), chemotherapy was started within two weeks after diagnosis.

**Figure 1:**
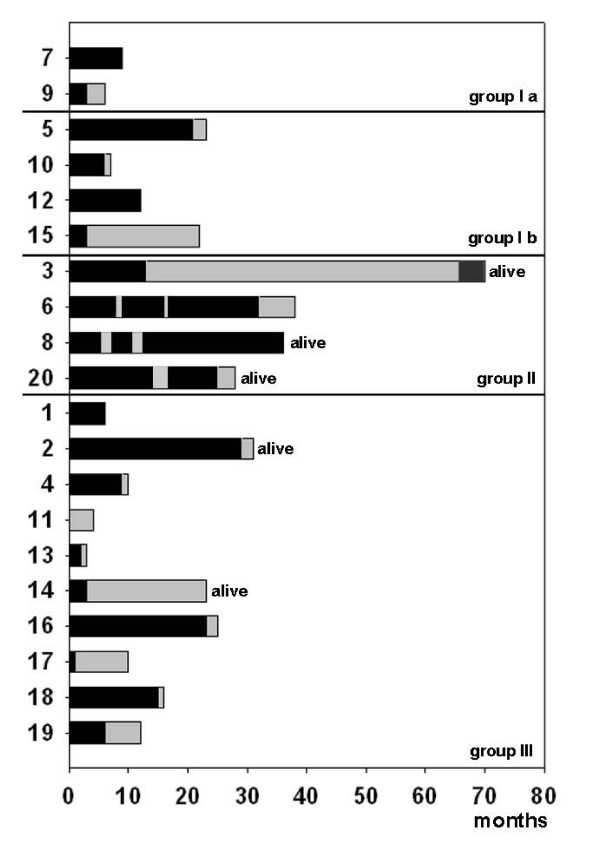
**Time of chemotherapy administration (black bars), relative to individual observation time**. The grey bars mark the time without chemotherapy. Group numbers refer to the response of the primary, described in Table 2.

Chemotherapy was given over, on average, 13 months (SD ± 11) with a mean observation time of 14 months (Figure 1).

### Safety and toxicity

In most of the patients, the treatment was well-tolerated. Treatment-induced grade 3 and 4 toxicities which resulted in a reduction of treatment or a change of the treatment regimen included leukopenia (3 patients), anemia (1 patient), infection (2 patients), diarrhea (2 patients), polyneuropathy (2 patients), and hand-foot syndrome (1 patient). Thrombembolic complications were suspected in two patients. In one of these patients bowel perforation occurred, making emergency operation necessary (Pat. 10). At the time, the patient had received a combination therapy including bevacizumab. There was no clear relation with endoscopic treatment, as the perforation was noted 5 weeks after the last colonoscopy. In all patients receiving bevacizumab, no bleeding complication occurred. More than one adverse event was found in 3 patients. 11 patients (55%) did not show treatment-relevant side effects.

However, nine patients received less than 6 months of chemotherapy as after an initial general improvement, clinical condition declined due to general disease progression.

### Observation of clinical symptoms

Initial symptoms were reported in 95% of the evaluable patients (17/18). Local symptoms of the primary were reported in 83% (15/18). Initial symptoms of the primary tumor included bleeding in 44% (8/18) of the patients, pain in 44% (8/18), and irregular stools in 50% (9/18).

Accompanying symptoms at presentation were reported in 78% (14/18) of these patients. They included weight loss (56%, 10/18), night sweat (39%, 7/18), and fatigue (28%, 5/18).

At the first follow-up staging visit after 3 to 4 months of therapy, both, local as well as general symptoms had improved. As for local symptoms, bleeding was only reported in 5% (1/18) of the patients. This patient had received acetyl salicylic acid. Pain was recorded in 11% (2/18), and irregular stools in 11% (2/18) of the patients.

Accompanying symptoms at follow-up were mainly reduced to fatigue (28%, 5/18), which also might be a side effect of chemotherapy and/or accompanying anemia. Further weight loss was only reported in one patient (5%, 1/18) and night sweat in another patient (5%, 1/18).

### Local behaviour of the primary

**Figure 2:**
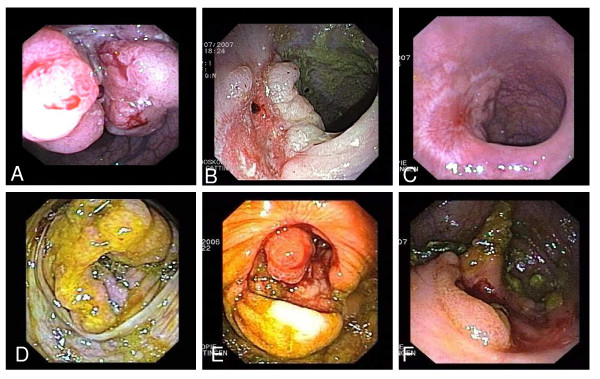
**Endoscopic images of the behaviour of a rectum carcinoma (A-C) and of another case with a carcinoma at the ileocoecal valve (D-F) before and during therapy**. A and D) at diagnosis; B and E) after endoscopic tumor debulking and three months, C) after 6 months, and F) after 15 months of chemotherapy.

**Figure 3:**
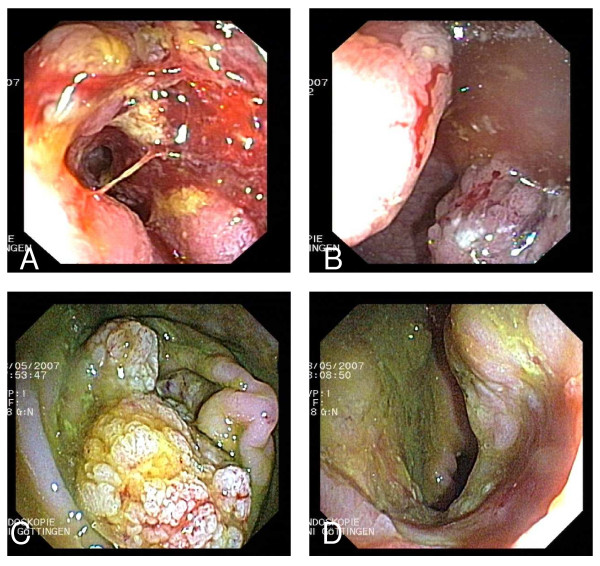
**Left panel: endoscopic images of a right-sided adenocarcinoma at 90 cm ab ano**. Right panel: endoscopic images of a second carcinoma at 15 cm ab ano in the same patient. A) and B) at diagnosis, C) regression of the carcinoma of the right-sided after three months of chemotherapy. D) left-sided carcinoma after three months of chemotherapy.

In all patients, stable disease or regression of the primary tumor during therapy was observed (Table 2, Figure 2 and 3). This response was an early event, and was already seen at the first endoscopic inspection, in some cases as early as after one month of therapy. None of the patients had to be operated because of complications due to uncontrolled growth of the primary tumor.

**Table 2:** Individual local tumor response.

Macroscopic tumor response		Patient	Initial tumor size (% tumor of intestinal lumen)	after 3–4 months of therapy	best response
**Complete luminal response of the primary** **(group I)**	**Normal mucosa (Ia)**	Pat. 07	85%	30%	30%
		Pat. 09	85%	0%	0%

		**death**	related to metastasis	n = 1	0 alive
			related to other causes	n = 1	

	**Adenomatous tissue (Ib)**	Pat. 05*	85%	70%	30%
		Pat. 10*	R: 85%/L: 50%	R: 60%/L:	R: 60%/L: OP
		Pat. 12	85%	30%	5%
		Pat. 15*	95%	20%	40%
				85%	

		**death**	related to metastasis	n = 3	0 alive
			related to other causes	n = 1	

**Relapse after therapeutic break (group II)**	**Initially no tumor cells at biopsy**	Pat. 03	80%	60%	0%
		Pat. 06*	85%	30%	30%
		Pat. 08*	100%	85%	55%
		Pat. 20*	75%	25%	25%

		**death**	related to metastasis	n = 1	3 alive
			related to other causes	n = 0	

**Partial macros-copic response of the primary** **(group III)**	**Histologically confirmed tumor tissue**	Pat. 01*	85%	85%	85%
		Pat. 02*	90%	85%	70%
		Pat. 04*	85%	80%	80%
		Pat. 11*	85%	75%	75%
		Pat. 13*	100%	85%	85%
		Pat. 14	60%	40%	OP
		Pat. 16*	60%	30%	20%
		Pat. 17*	100%	85%	85%
		Pat. 18*	90%	85%	85%
		Pat. 19*	80%	70%	65%

		**death**	related to metastasis	n = 8	2 alive
			related to other causes	n = 0	

* incomplete = reduced amount of therapy or therapeutic break

Complete initial luminal response of the primary CRC, with no malign tumor material at biopsy, was found in 10/20 (50%) of the cases. In four patients, the luminal side of the intestine was completely intact with no adenomatous tissue visible during endoscopy. Later-on, two of these patients showed local tumor relapse after cessation of systemic therapy: One patient 53 months after therapy interruption. This patient had initially been considered as cured (Pat. 3). The other patient (Pat. 20) showed luminal tumor recurrence 2.5 months after therapy interruption due to pneumonia with sepsis.

In the other six patients, only adenomatous tissue was found histologically. Examples are given in Figure 2C and 2F, and Figure 3C. After interruption of the therapy at the patients' request for a break, two of these patients showed local tumor relapse as well (Pat. 6 and 8).

The 10 patients with complete initial luminal response are summarized into two groups. Group I: Complete response of the primary with I a, normal mucosa; and I b, adenomatous tissue (table 2). The patients who relapsed after therapy interruption are in group II.

In the remaining 10 patients (50%), partial luminal response or stable disease of the primary was observed (table 2). In these cases, the primary CRC remained macroscopically apparent. These patients are summarized as group III.

Tumor debulking was performed in patients with initial symptoms due to the primary. As, at that time, we could not predict what the effect of chemotherapy would be, in this group (group III) "prophylactic"endoscopic treatment was repeated to prevent possible recurring luminal obstruction, especially in those patients who were not able to complete chemotherapy cycles or in whom the dosage had to be reduced. This was the case in six patients.

In one of the patients, the primary carcinoma as well as the tumor in the lung was operated after local tumor regression (Pat. 14).

### Overall survival and time-to-progression of the primary

At the cut-off date, the observation time of the study was 6 years and 9 months. Median follow-up time was 14 months (range 3–71 months), as 15 patients had died during the observation period. In none of these patients was death related to local complications or growth of the primary.

In group I and II, five out of 10 patients (50%) died of metastatic disease progression (Table 2). Mean overall survival time (OS) for these groups taken together was 25 months (median 22.5, range 6 to 71 months).

In group III, which includes patients with macroscopic regression of the primary with histologically confirmed tumor cells at the end of the observation time, eight out of ten patients (80%) died of metastatic disease progression. Mean OS in this group was 14 months (median 11, range 3 to 31 months).

**Figure 4:**
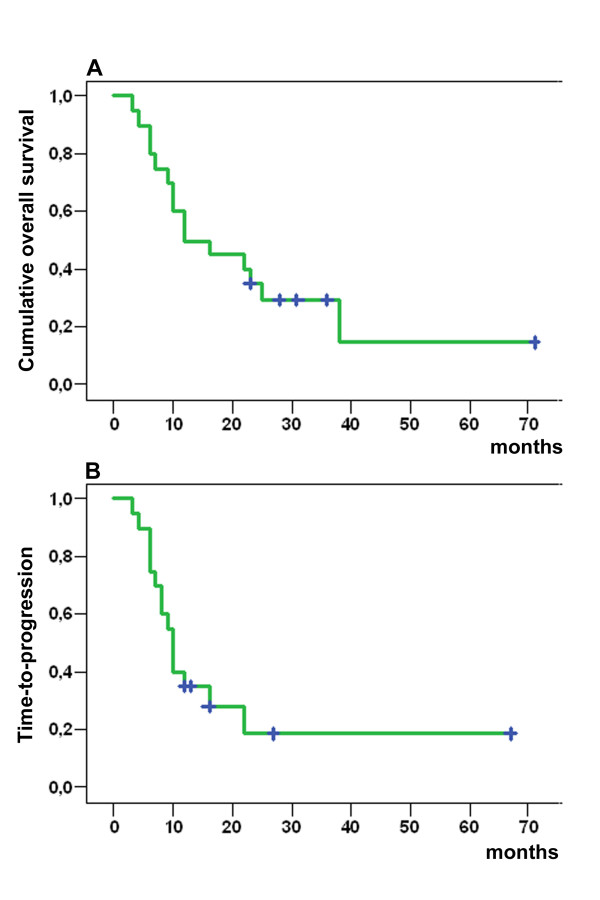
**A) Cumulative overall survival (months) of all patients (n = 20)**. B) Time-to-progression of the primary tumor (n = 20). The patients, which were alive at the end of the study are marked with a cross. Survival curves were generated using the Kaplan-Meier method.

OS over the whole patient collective was, on average, 19.6 months (median 14, range 3 to 71 months) (Figure 4a). It was significantly improved when 100% of the calculated chemotherapy dosage could be given and chemotherapy cycles were completed (Table 2). Time-to-progression (TTP) of the primary was in the mean 13.6 months (median 10, range 3–67 months, Figure 4b). The subgroup-analysis of group II showed a TTP of in the mean 29 months (median 20.5, range 8 to 67 months).

In fact, under ongoing chemotherapy with completed chemotherapy cycles, progress was observed only for the metastases and not for the primary. Additional secondary metastasis to the lung, the peritoneum or the bone marrow under ongoing chemotherapy occurred in eight of the 20 patients (40%).

## Discussion

For most patients with CRC, surgical resection with or without chemo(-radio)therapy is the standard treatment approach. The treatment of surgically non-curable metastasis of colorectal cancer is palliative chemotherapy [28,29].

Taking into account that in many cases bleeding of the tumor or obstructive disturbances can now be treated endoscopically [25,27,30], the indication for surgery of the primary tumor in patients with unresectable metastatic CRC appears more controversial [13,21,31]. The fact that after primary-directed-surgery in stage IV disease a significant proportion of patients do not get palliative chemotherapy; e.g. in the study of Temple et al. 40% of the patients above 65 years of age did not receive chemotherapy after surgery of the primary tumor [32], argues against initial surgery of the primary. Supporting this argument, several retrospective studies have shown that first-line chemotherapy is safe [21] and more effective than it was before 2002 [31]. In addition, the rate of gastrointestinal complications related to the unresected primary is low and comparable to those observed in resected patients [14,21]. When surgical resection of the primary was compared to conservative treatment in the setting of incurable stage IV disease, no survival advantage was gained by the resection of an asymptomatic primary [21]. It is understood that most of these patients die of their systemic disease before the development of a major complication related to an intact primary lesion [21,33].

Although the number of patients in our study is limited, it represents a further attempt to show that chemotherapeutics combined with interventional endoscopy avoid the need for resection of the primary in unresectable stage IV CRC, even when the primary is symptomatic (obstructive or bleeding). From our study we are unable to comment on the relative effectiveness of chemotherapy or endoscopic debulking in isolation. However, interventional endoscopy might not be necessary in asymptomatic patients under effective chemotherapy.

The survival rate of our patients is similar to that of other studies in which the primary tumor had been surgically resected and where subsequent chemotherapy was possible [10]. We are aware that the results of our study should be considered as tentative and hopefully will encourage future larger studies. In mono-centric studies, large numbers cannot be obtained for this indication.

Patients' characteristics in our study were comparable to other studies performed in a patient collective with metastasizing CRC as regards the location of the primary and the site of metastasis [8,12]. However, they were distinct for age and performance status at the beginning of the study. The median age in our study was, at 67 years, older than the 65 years reported in other studies. Two Patients were above 80, six patients were between 70 and 80 years old. Six of the ten patients with macroscopic tumor regression (group III) were older than 70, and could not receive complete chemotherapy courses. These patients are also those who might not receive chemotherapy after primary-directed surgery [32]. Old age is a negative prognostic factor for general outcome, making preventive medical check-ups inevitable. Early administration of 100% of the calculated chemotherapy dose with completed chemotherapy cycles was associated with a significantly better OS and TTP of the primary in advanced disease.

## Conclusion

In the present study, combined endoscopic and pharmacological therapy was found to be effective and safe not only in asymptomatic, but also in symptomatic advanced stage IV CRC.

Our treatment improved both the initial symptoms of all patients and reduced or stabilized the size of the primary tumor. We therefore confirm and further expand the data by Chau et al., and others who reported rapid symptomatic response after neoadjuvant treatment in patients with colorectal cancer [13,31,34]. The survival rate of our patients is similar to that of other studies in which the primary tumor had been operated [10]. Furthermore, this study shows that anti-cancer drugs combined with endoscopic therapy may achieve a local response in the majority of the patients: The primary tumor seems to respond more effectively to chemotherapy than its metastases.

## Abbreviations

CEA: (carcinoembryonic antigen); CRC: (colorectal cancer); ECOG: (Eastern Cooperative Oncology Group Performance Status); OS: (overall survival); TTP: (time-to-progression).

## Competing interests

The authors declare that they have no competing interests.

## Authors' contributions

SC has prepared the manuscript and is responsible for the data collection. DH supervised the chemotherapy of the patients and collected the data for chemotherapy. She performed the Kaplan Meier analysis of the treatment outcome. TM did some of the documentation of the endoscopic images of the tumors. TA, JGS, HS and GR did the endoscopic tumor debulking and were responsible for the treatment choice of the patients. LF performed the histopathological analysis of the tumors. GR contributed to the main ideas of this work, selected the patients and helped to realize the paper. All authors have read and approved the manuscript.

## Ackowledgement

We are thankful to the collegues and the nurses of the department of gastroenterology and endocrinology who participated in the treatment of the patients. We wish to thank all the patients for allowing us to summarize the clinical data for publication.

## Pre-publication history

The pre-publication history for this paper can be accessed here:

http://www.biomedcentral.com/1471-2407/9/218/prepub
